# The importance of health clubs in asthma management

**DOI:** 10.4314/gmj.v55i4.5

**Published:** 2021-12

**Authors:** Chinonyelum T Ezeonu, Uzoma V Asiegbu, Odirichi Andrew, Chinwe I Joe-Akunne, Lilian N Nwobashi

**Affiliations:** 1 Department of Pediatrics, Alex Ekwueme Federal University Teaching Hospital, Abakaliki, Nigeria; 2 Institute of Child Health, Alex Ekwueme Federal University Teaching Hospital, Abakaliki, Nigeria; 3 Department of Pediatrics, Ebonyi State University, Abakaliki, Nigeria

**Keywords:** social support, health club, children, asthma

## Abstract

**Objectives:**

To describe the importance of Health clubs in Asthma management in Alex Ekwueme Federal University Teaching Hospital, Abakaliki, Ebonyi state.

**Design:**

a cross-sectional qualitative study using focus group discussions (FGD) and Key informant interviews (KII)

**Setting:**

the study was conducted from June to July 2019 at the Pulmonology Unit, Department of Pediatrics, Alex Ekwueme Federal University Teaching Hospital, Abakaliki, a tertiary health facility in Ebonyi State, Nigeria

**Participants:**

15 out of 25 caregivers of our pediatric asthma clients who attend the Asthma club and nine key informants drawn from club members and some care givers.

**Data Collection/Intervention:**

With informed consent, data were collected from participants using recorded interviews

**Statistical analysis/Main outcome measure:**

This was done using the descriptive phenomenological psychological by sorting out each description's explanation and separating them into inferred meaning units for analyses and discussion for easier analysis.

**Results:**

Out of 25 caregivers invited, 15 participated in the FGD, all were females aged 32–57 years. For the KII, two out of nine participants were males, three were parents (adults), whereas six were children, 9 to 16 years. Generally, most respondents expressed that the Asthma club improved their sense of well-being y providing a better understanding of the illness, and skills for its management.

**Conclusion:**

The asthma club provides informational and affirmational supportive value to clients. We encourage as many centres as possible to raise an asthma club for more effective health communication and asthma care.

**Funding:**

none declared

## Introduction

Asthma is an episodic yet chronic airway disease that places a heavy socioeconomic, emotional, and psychological burden on patients and their families. It often results in episodic airflow obstruction.^1^ The prevalence of asthma increases with communities' urbanisation and lifestyle.^2^ Globally, over 300 million persons are affected by this non-communicable airway disease.^3^ It is a chronic disease that requires long term medications and lifestyle adjustments in management. It affects all age groups, children inclusive. Amongst children, asthma is a known cause of school absenteeism,^4^ which can adversely affect school performance and the psychosocial function of the child.^5^ There are no doubts that some children with such chronic disorder have been challenged with such questions as ‘when will I be allowed to play like other children? ‘Why can't I be allowed to go to school without this inhaler and a cardigan like other kids?’ And their parents would ask, ‘Will this child ever outgrow this Asthma?’ ‘How long will this child be on inhaled steroids?

These emotional questions deserve to be answered, but the real fact is that the restricted clinic visit times might not be sufficient to provide them with the answers and much needed support. Emotional discomfort creates negative treatment bias and poor adherence to prescriptions. Poor understanding of the medication regimen, substandard education on symptom recognition and environmental triggers, rejection of the diagnosis, and a lack of support or understanding within the community contribute to poor adherence to medication. 6 Masoli et al. noted that problems amongst asthma patients include difficulties in access or use of health care, poor adherence to treatment regimens^3^ and lack of self-management skills.^7^, ^8^ Parental perceptions and practices are also crucial for improving asthma outcomes in children.^9^

The management of asthma must accommodate the socioeconomic, psychosocial and emotional factors that affect asthma perception. Indeed, to answer these questions, achieve better patient cooperation and reduce poor adherence to medications, health services must be integrated and people centered.^10^

Informal caregivers can assist asthma patients in obtaining, understanding, and utilising basic health information needed for appropriate health decisions and self-management.^11^, ^12^ Asthma treatment guidelines now recognise peer-led and peer support education as ways to complement the usual clinician-based care to address poor adherence among adolescents. 13, 14

Peer support education refers to providing emotional, appraisal and informational assistance by a member who possesses experiential knowledge of a specific behaviour or stressor and similar characteristics as the target population”.^15^ This differs slightly from lay-led interventions where people may not necessarily provide emotional, appraisal and informational assistance with asthma or other chronic conditions. In other words, peers in this context are not medically trained but are similar to the target population in terms of age and asthma diagnosis or diagnosis of a different long-term condition.

People suffering from chronic disorders must benefit from various forms of social support.^16^ Social support can be classified into three categories, emotional, informational, and affirmational. Emotional support enhances self-esteem and self-efficacy by exchanging personal difficulties, empathy, and reassurance with people in similar situations. Informational support provides relevant information and advice that may help people engage in more effective self-management, whereas “affirmational” support helps people generate a positive outlook to reduce the stigma of a long-term health condition. 15

The establishment of self-management and lay-led programmes in health care is expected to allow people with chronic diseases to have access to some of these social support opportunities to develop the confidence, knowledge and skills to manage their conditions better, and thereby gain a greater measure of control and independence to enhance their quality of life.” 17 It is necessary to note that a person's belief or perception about his health and treatment influences his medicationtaking behaviour.^18^ Any intervention capable of changing an individual's belief is known to improve compliance with medications and health outcome.^19^

Health clubs can be an avenue for behavioural change communication, establishing self-management and providing social support. They may be formed as lay-led or peer-led or health care provider-led initiatives. Activities offered by such clubs enable members to learn valuable skills for health promotion. Members of the health club are made to have a sense of ownership, as they are expected to contribute to the organisation's planning, maintenance, and success.

Experience from the practice as a clinician in our locality has revealed some negative attitudes of clients towards the diagnosis of asthma. Most of them perceived it as a curse that must be rejected, arguing that there is no such history in their family. These arguments were mostly borne out of ignorance and potentially fostered underutilisation of health services, poor medication compliance, and loss of follow-up. Consequently, late presentations to the hospital in acute severe asthma exacerbation were more often the norm.

Knowing that poorly controlled asthma ultimately affects the client's quality of life, we sought to find a way to demystify asthma and increase their awareness and compliance to the asthma control therapy. An asthma club, known as the ‘Breathers' Club’ was set up in 2016 as a non-governmental organisation made up of asthmatic children and people who live/work with asthmatic children. This paper attempts to describe the effect of the Asthma Club in our facility on our asthma clients using a qualitative approach.

It is a pediatric asthma club domiciled in the tertiary health facility, Alex Ekwueme Federal University Teaching Hospital, Abakaliki. The objectives of the Club are to provide basic information about asthma in an informal and friendly setting; to create a support system where children with asthma and parents of asthma patients can interact with each other and share their experiences; to demystify asthma increase compliance to medications.

The Breathers' Club has both human and material resources to help in their activities in terms of input and activities. The human resources consist of volunteers, made up of paediatricians and paediatricians-in-training, nurses, health educators, parents, and very enthusiastic child/adolescent members.

The officials of the Club are a mix of health workers, adolescent members, and parents. Membership of the Club is not exclusive to Asthma patients but includes parents, friends, school teachers and any other person interested in learning about asthma.

Material resources such as funds, free spacer devices, peak flow meters and drugs at subsidised rates were occasionally sourced and obtained from donors, pharmaceutical companies, and the hospital management. These resources give the Club the capacity to organise health talks, hold discussions with parents and their children, organise educational games, debates, quiz competitions and demonstrate the appropriate technique for using inhalers, spacer devices and peak flow meters. We had a space within the Institute of Child Health, Alex Ekwueme Federal University Teaching Hospital, used for most club activities. Through interactive sessions, the Club tries to address most of the emotional needs of her clients. It offers a periodic gathering of paediatric asthma clients in an informal manner, where patients are attended to in a more relaxed mood than the usual time-limited clinic visits. The Club offers periodic updates or seminars on asthma issues to be delivered to members by health workers, laypersons, or peers in very simple terms. Major activities of the Club each year include the celebration of the World Asthma Day annually, holding Kids-Exclusive forum every August/September Holidays and then Parents forum in October/November. These are information-packed activities of group discussions, drama, fun-filled activities such as spelling competitions, quiz etc. Some of our activities are peer-led, especially the Kidsexclusive forum. Others are lay-led, as in the Parents forum, which offers an opportunity for parents/caregivers to discuss their strengths and weaknesses in managing a child with asthma. There, they share their experiences, educate one another while the Pediatricians and other club officials moderate the discussion.

The Breathers' Club has existed for about three years now. So it became necessary to evaluate its relevance and capacity to achieve its objectives using a qualitative research method via focus group discussion and key informant interviews with the target clients. To the best of our knowledge and search, there is no available literature on the presence and evaluation of the benefits of Asthma Health Clubs for children in most parts of our country, Nigeria, hence the need to do this work.

## Methods

### Study site

This qualitative study was conducted from June to July 2019 at Alex Ekwueme Federal University Teaching Hospital, Abakaliki, (AE-FUTHA), a tertiary health facility in Abakaliki, the capital city of Ebonyi State, southeast Nigeria. It is a relatively dry and dusty area, especially around October to April. It is a zone of mineral resources such as limestone and lead; therefore, many mining works and stone crushing go on all year round.

AE-FUTHA has functioned as a referral centre and serves as a primary and secondary health centre for many in its environs. It has a Pediatric Pulmonology clinic that provides services for respiratory illnesses, including Asthma, for over 56 clients. The clinic initiated the creation of the Asthma club. Thirty-five asthma clients aged between 7yrs and 18yrs were registered for membership with the Club for three years. A total of 25 adults registered with or without their children.

### The study participants

All the parents of clients registered with the asthma club were eligible to participate in the Focus group discussion. However, we took cognisance of those who were more regular in club activities, especially the Parents' forum. Those willing and able to participate were included in the study. Child/adolescent members of the Asthma club and some of their parents who had regularly attended the Club's activities were purposively selected as key informants.

### Study design

It was a cross-sectional qualitative study method useful in health science research for programme evaluation.^20^

### Data collection

Phone call invitations and reminder SMS for a Focus group discussion were sent to all 25 caregivers of our pediatric asthma clients attending the Club, using their names/phone numbers from the club register. Only 15 persons who gave their consent turned up and participated in the round table discussion. The researchers prepared a list of questions to be administered. The participants' biodata were documented at the beginning of the Focus Group Discussions (FGD). Some basic rules to guide the discussions were laid out, such as no crowded answers, giving each other a chance to speak and express his/her opinion without interruptions, and not speaking for too long to allow others to speak. The participants were split into two groups of seven and eight, each with a moderator and recorder. The facilitators of the discussion were already trained and conversant with their role as moderators of the discussion.

We later conducted key informant interviews with some of our Asthmatic clients and some parents. Our key informants were purposively selected from the few clients who appear most knowledgeable of the Club's activities by being regular in meetings. We selected some adolescents, younger children, and a few parents just for a variety of perspectives. This was necessary to evaluate the Club's effect since the authors considered that there might be no in-depth response from a member who rarely attends.

The key informant interviews (KII) were performed by a team of two trained facilitators, a medical doctor (club member/facilitator) and a Public relations officer (not a member of the Club), with skill in conversationally asking questions, maintaining appropriate dialogue. We also recruited a skilled recorder, who recorded every conversation with his voice recorder to avoid missing anything. Anonymity was ensured by coding the names of the respondents, and some of their statements were reported verbatim.

### Data analysis

This was done using the descriptive phenomenological psychological method of qualitative data analysis by Giorgi et al.^21^. Data were obtained by sorting out each description's meaning or explanation and separating them into inferred meaning units for analyses and discussion. The responses from Focus group discussions were harmonised as group opinions and presented in tables. For the KII, some salient statements were reported verbatim.

### Ethical consideration

Consent to do this work was obtained from the Ebonyi State University Research Ethics Committee on May 12 2019 with reference number EBSU/DRIC/UREC/Vol.05/073. Consent was duly obtained from participants after explaining the study method and assuring them of confidentiality and anonymity of their reports.

## Results

Fifteen parents/caregivers, out of twenty-five invitees turned up for the focus group discussion. Most of the absentees gave excuses due to the exigencies of their various duties. All of those that attended were females, with the age range 32 – 57 yrs (mean – 39.1 years). None of the males turned up. Eight out of fifteen (60%) had tertiary education, whereas the rest (40%) had secondary education as the highest level.

### Key informant interview of some members of the Breathers' Club

For the KII, two out of nine participants were males, three were parents (adults), whereas six were children, with age range of 9–16 years. Some of their comments/responses are as shown below. The aim was to assess their impression about the Breathers' Club.

The interview guide consisted of four questions as follows: i) How satisfied are you with the programmes of the Breathers' Club? ii) What are your perceived benefits of joining the Asthma club? iii) To what extent can you attribute some behavioral changes, if any, to the Asthma club? iv) Are there suggested areas that need improvement in the Club?

#### 1) How satisfied are you with the programmes of the Breathers' Club?

This question revealed the degrees of satisfaction amongst some of our clients. A 16-year-old, who was one of the founding members of the Club, and who joined the Club at the age of 13yrs said “Breathers' *Club has been wonderful. I am more confident* .... Another 16yr old member said, *I have learnt a lot from the Club*.... A 12 yr old respondent who used to have frequent flare ups said “*I am quite comfortable now. I can control my asthma better*... m*y last attack was 4 months ago .....*”

#### ii) What are your perceived benefits of joining the Asthma club?

Bearing in mind the various objectives of the Club which includes empowering clients with health information on asthma and creating a support system, the benefits of the Club were sought from some of the members. One of them said “I can talk to friends about asthma”. A mother of two asthma clients of the Club, said “*My children and I have benefited a lot, since they started attending the club functions, they now know their asthma triggers and try to avoid them....*” Another informant said with excitement, *“I can confidently speak to my friends at school about asthma....”*

*I have made some friends in the asthma club*” said an 11-year-old.

#### iii) To what extent can you attribute some behavioral changes, if any, to the Asthma club?

One of the members, formerly with poor adherence to medication, reported that she now takes her controller medications as when due “*I have less frequent attacks. My breathing is better and I have better confidence that I can control my asthma*.

The mother of another child who previously had difficulties accepting asthma as a diagnosis responded, *“I have personally been exposed to the highlights of asthma management through club lectures. It was great for me when I was called to give a talk to other parents during the Club's Parents' forum*.

Another mother whose 16 year old son has not been complying with his medications said *“I am glad that I have attended the club programmes with him and I personally learnt a lot...” I am the one encouraging him the more to take his medications*”

Another mother reported that her kids are beginning to manage themselves. She reported that her kids say things like “*Mummy I need new bed sheets, I can't use that old one, it is now dusty”*

Our 9 year old client said “I learnt how to use the peak flow meter and *I now use my inhaler better with a spacer device”*. Another informant, an 11yr old said *“I now use my peak flow meter to check myself”.*

A similar behavioral change was reported by a 16yr old female informant who said “*I now use my inhaler better because I was not actually using it well before now. I now have a peak flow meter courtesy of the club*...”. She also said ‘*I have also learnt that asthma cannot stop me from being whom I want to be*, ...” On the other hand, our 16 year old male informant still found it difficult to accept Asthma and is not yet complying well with his medications and necessary precautions.

### Are there suggested areas that need improvement in the Club?

Most of our respondents suggested better publicity on the existence of the Club. One of the adolescent respondents observed that schools were very important areas that the Club had not reached extensively. She said..... *“Breathers' Club should do more outreaches to many schools, educating them about asthma and showing them people who have benefitted from the club”.*

## Discussion

In this report, we have tried to find out to what extent the objectives of the Asthma club are met. to provide basic information about asthma in an informal and friendly setting. The FGD and KII revealed acquisition of useful, basic information about asthma that supports selfmanagement and parental partnership in asthma care. From the foregoing, it appears that the Club is providing informational support to her clients necessary for more effective self-care.^15^

The setting of the Club provides opportunities for informal and in-depth discussions with clients which may mimic counseling services. Lee et al noted that asthma patients were more satisfied with counselling that discussed and explained the features and management of asthma.^22^ Opportunities for informal interaction with healthcare providers is offered through the Club, creating a chance for asthma clients to ask important questions, to enhance self-management.^23^

An expression of confidence in talking to friends about asthma has shown the capacity of the Club to provide affirmational support, which potentially reduces stigma.^15^This also may have explained the expression from one of the key informants that asthma cannot stop her from being who she wanted to be. Health clubs have the potential to improve a sense of well-being amongst participants especially if it provides better understanding of an illness and skills for its management, in a stigma free environment. Some of our key informants categorically stated that they felt better and more confident in managing their asthma following lessons learnt from the asthma club. This effect has been demonstrated in a study by Settergren et al., on the importance of support groups amongst children living with HIV in Haiti.^24^ This feeling of well-being is a shared benefit between the asthma patient and the parent/caregiver. A report from one of our parent key informants that she now encourages her son to take his medication suggests that better understanding may influence parents' enthusiasm to support or seek adequate care. Increased social support for those living with asthma, specifically from parents or peers, has been reported to be associated with maintenance of a healthy lifestyle among adolescents, which may serve to reduce their exposure to unhealthy behaviours likely to exacerbate their symptoms.^25^, ^26^

Most of our key informants were adolescents. We found it interesting to hear their reports of positive disposition to asthma following lessons and experiences gathered from the Club. Although this study did not objectively assess medication adherence amongst our clients, a report of improved compliance to medication from some of our key informants was quite encouraging. In a study by Foot et al on asthma medication adherence, it was found that improving patient understanding of their illness positively enhances their compliance with treatment.^27^

It is important to note here that adolescence is an important time to have established good self-management behaviours that will see them through to adulthood. It is a time of peer pressure either positive or negative. Provision of opportunities that enhance their understanding and skills to manage their problems helps improve their positive disposition to face the social and psychological challenges of their time. Excitingly, some of our adolescent key informants expressed positively their ability to control their asthma and their confidence and ability to speak up to others about asthma.

Some of the expressions made by club members and/their caregivers indicated the value of learning in an informal club setting. Reporting less frequent attacks and improved skills in using the inhaler and devices such as spacers, peak flow meters buttress the above point.

Physicians and health care providers owe their asthma clients a duty of appropriate communication on management procedures. Shum et al noted that lack of sufficient instructions from physicians, language/communication barriers, lack of skills on how to use inhalers, and high medication costs were some of the barriers to asthma medication adherence.^28^ The asthma club particularly worked towards closing such gaps in order to achieve better patient cooperation. On the other hand we obtained useful information from our respondents on areas for improvement on our interventions. Suggestions by our informants and discussants to create wider publicity for the Club gives us the impression that they would want many more people to learn what they may have learnt about asthma and its care. This suggestion is worthwhile since dissemination of asthma related information to the general public using the mass media may help to demystify asthma, inform the community and achieve better control of asthma. 29

There are no doubts that our study had several limitations. It has not compared the effects of not being a member of the Club with that of club members, hence we cannot conclusively attribute the reported benefits to the club program. Another limitation is the small numbers and lack of gender balance. Purposive selection of regular participants may not be without bias hence the need for larger numbers and comparators. This will be considered in future studies.

## Conclusion

In this report we describe the establishment of an Asthma club in our health facility as a support group, its short term effect on asthmatic patients and its potential role in behavioural change communication for children living with asthma and their caregivers. It appears that this Club provides informational and affirmational supportive value to clients, therefore we encourage as many centers as possible to raise an asthma club for more effective health communication and asthma care.

## Figures and Tables

**Table 1:** Information obtained from the Focus group discussion with parents of our clients in the Breathers' Club

Questions	Summarised Answers	Inference
What do you now know about Asthma?	Asthma is an allergic condition that makes breathing faster. It is generally believed to have triggers such as smoke.	They have some basic information about asthma
What are the triggers of asthma?	Commonly mentioned triggers were exposure to Cats, Dust, Excitement, Perfumes, Smoke, Cold water/drinks, Poultry, Fried foods.	They have a grasp of possible triggers
What has been your feelings about Asthma?	They generally felt that Asthma is dangerous enough to kill a child. They believed it can put a parent into trouble. It restricts parents/child from cooking or eating what they like.	They are very cautious about asthma
Have you learnt how to use an inhaler?	12 out of 15 (80%) had learnt how to use an inhaler and so could monitor their kids	They have a grasp on the importance of inhaler technique
Do you worry about your asthmatic child? What worries you?	They worried about their children's safety while at school especially in a boarding school. They worry about the continuous use of drugs by the children for fear of side effects	They are still scared about asthmatic attacks and also daily use of drugs eg steroids
Have you been regularly attending Breathers' club programs? If yes has it been useful?	13 out of 15 (86.7%) said yes and they feel it has been useful.	They find the Club useful
How has the Club helped?	Most have learnt how inhalers and peak flow meters are to be used and have gained materials such as peak flow meters, recording chats (free of charge) for managing asthma. They also got encouragement from interacting with other parents	The Club provides forum and materials for learning by interaction
How can we improve the club activities?	They suggested more advocacy and publicity to increase awareness to the general public. Link up with other Asthma support groups. Go on outreaches to schools to create awareness. Expand outside AEFUTHA to other hospitals like general hospitals. Equipping schools with first aid materials, and teaching school nurses how to detect asthma symptoms and apply first aid before referral.	There is need for wider publicity on Asthma

**Table 2:** A summarised tabular presentation of the Key informant's responses

Key Question	Verbatim Response	Meaning
i) How satisfied are you with the programmes of the Breathers' Club?	Breathers' Club has been wonderful... I am more confident (16yr old) “I have learnt a lot from the club” (16yr old) “I can control my asthma better ... my last attack was 4 months ago... (12yr old)	Members found it satisfactory
ii) What are your perceived benefits of joining the Asthma club?	“I can talk to friends about asthma” (16yr old) “My children now know their asthma triggers and try to avoid them” “I have made some friends in the asthma club” (11yr old)	They gained useful information, confidence and new friends
iii) To what extent can you attribute some behavioral changes, if any, to the Asthma club?	I now understand why ...... I take my controller medications as when due. “I am glad that I attended the club programmes with him ... I am now the one encouraging him the more to take his medications” (mother of member) My kids are beginning to manage themselves, they would say.... "Mummy I need new bed sheets, I can't use that old one, it is now dusty” (mother of members) I now use my inhaler better with a spacer device” (9yr old). "I now use my peak flow meter to check myself” (11 yr old). “through the club I have also learnt that asthma cannot stop me from being whom I want to be” (16yr old)	Positive behavioral changes were gained
iv) Are there suggested areas that need improvement in the Club?	Breathers' Club should do more outreaches to many schools...	Need for wider publicity.
